# Hepatic Transcriptome Analysis of Hepatitis C Virus Infection in Chimpanzees Defines Unique Gene Expression Patterns Associated with Viral Clearance

**DOI:** 10.1371/journal.pone.0003442

**Published:** 2008-10-17

**Authors:** Santosh Nanda, Michael B. Havert, Gloria M. Calderón, Michael Thomson, Christian Jacobson, Daniel Kastner, T. Jake Liang

**Affiliations:** 1 Liver Diseases Branch, NIDDK, NIH, Bethesda, Maryland, United States of America; 2 Genetics and Genomics Branch, NIAMS, NIH, Bethesda, Maryland, United States of America; 3 CBER, FDA, Bethesda, Maryland, United States of America; 4 Virology Department, GlaxoSmithKline, Research Triangle Park, North Carolina, United States of America; 5 Departments of Biology and Mechanical & Mechatronics Engineering, University of Waterloo, Waterloo, Ontario, Canada; Beijing Institute of Infectious Diseases, China

## Abstract

Hepatitis C virus infection leads to a high rate of chronicity. Mechanisms of viral clearance and persistence are still poorly understood. In this study, hepatic gene expression analysis was performed to identify any molecular signature associated with the outcome of hepatitis C virus (HCV) infection in chimpanzees. Acutely HCV-infected chimpanzees with self-limited infection or progression to chronicity were studied. Interferon stimulated genes were induced irrespective of the outcome of infection. Early induction of a set of genes associated with cell proliferation and immune activation was associated with subsequent viral clearance. Specifically, two of the genes: interleukin binding factor 3 (ILF3) and cytotoxic granule-associated RNA binding protein (TIA1), associated with robust T-cell response, were highly induced early in chimpanzees with self-limited infection. Up-regulation of genes associated with CD8+ T cell response was evident only during the clearance phase of the acute self-limited infection. The induction of these genes may represent an initial response of cellular injury and proliferation that successfully translates to a “danger signal” leading to induction of adaptive immunity to control viral infection. This primary difference in hepatic gene expression between self-limited and chronic infections supports the concept that successful activation of HCV-specific T-cell response is critical in clearance of acute HCV infection.

## Introduction

Since the identification of hepatitis C virus (HCV) in the late 1980s, HCV infection has been recognized as a growing public health problem in the world. It is estimated that about 200 million people are chronically infected [1]. No HCV vaccines are available to date and only a subset of patients respond to current interferon-based treatment [1]. Much research effort has been focused on understanding the mechanisms of infection, persistence and clearance of HCV. Studies on hepatic gene expression in chimpanzees and humans have revealed intriguing differences between acute resolving and chronic HCV infections [2]–[6]. In the chimpanzee model, HCV infection induces type I IFN response and activation of a large number of interferon stimulated genes (ISGs) in the liver [2], [6], [7]. However, hepatic induction of type I IFN occurred in all animals irrespective of the outcome of infection [6], [8]. Thus, type I IFN may restrict excessive viral replication in the early phase of infection but does not seem to play a major role in subsequent viral clearance. Similar to experimentally infected chimpanzees, gene expression analysis of percutaneous liver biopsies in HCV infected humans demonstrated increased ISG expression, suggesting an ongoing host cellular response to viral infection [4]. It is not well understood why HCV is not cleared from these individuals despite the activation of potent antiviral ISGs. It is possible that in these individuals with viral persistence, the cellular response to IFN is inefficient compared to those who clear the virus. Weak induction of the ISG antiviral state coupled with an ineffective cellular immune response could therefore promote chronicity [3].

Studies of temporal changes in gene expression are central to understanding viral clearance, persistence and hepatic injury in chronic HCV infection. The intrahepatic T cell response to HCV correlates with control of acute infection [7]. Cytokine and immunomodulatory genes, generally known to be chemotactic and/or stimulatory to various immune cells were observed to be induced within the first 6 to 8 weeks after infection [2]. Viral clearance is associated with a vigorous HCV-specific T-cell response in the liver with both cytotoxic and non-cytotoxic effector functions. However HCV persists in the majority of acutely infected patients. The mechanisms leading to the failure of HCV-specific T-cell response and viral persistence are highly complex and still not fully understood. Therefore it is important to compare the spectrum and magnitude of hepatic gene expression in individuals who spontaneously clear infection and those who progress to chronicity. In this study we examined the host response to HCV infection by applying gene expression profiling of serial liver biopsies during acute HCV infection and identified unique gene expression patterns that are associated with specific outcomes of infection.

## Results

### Hepatic gene expression during early stage of self-limited infection

To identify differentially expressed hepatic genes associated with early stages of HCV infection, a database of expression levels was generated and queried for genes whose expression was outside a calculated 99% confidence interval at different times. Using this confidence interval, 1% of genes with the greatest-fold change or least-fold change are defined as significantly up- or down-regulated (see Materials and Methods). For chimpanzee X0190 with self-limited infection, 347 genes were induced above the confidence interval (>1.9-fold change) at 4 weeks post-infection. Some of the induced genes were related to antiviral and/or type I interferon response, consistent with ongoing HCV infection. To refine our search and to further identify genes specifically associated with the early phase of infection, the database of expression levels for X0190 was queried again for genes above the confidence interval at two time points, weeks 4 and 6 post-infection (>1.9- and >2.1-fold induction), and below the confidence interval at weeks 13 and 40 (<2.2-, and <2.4-fold induction) when the chimpanzee had cleared the infection. By using these criteria, 22 genes were significantly up-regulated, 9 of which are known to be induced by type I interferon (Table 1). The remaining 13 genes are classified as Cellular Immune Response Related or Cell Growth/Signal Transduction Related.

**Table 1:** Genes induced during the early phase of self-limited and persistent infections.

Type I Interferon Response			Chimp X0190^a^				Chimp X0234				Chimp X0142							
Title	Clone description	UG cluster	wk 4	wk 6	wk 13	wk 40	wk 6	wk 8	wk 10	wk 12	wk 6	wk 8	wk 10	wk 12	wk 60	wk 68	wk 89	wk 101
G1P2	Interferon-stimulated protein (15 kDa)	Hs. 432233	***8.1***	***4.6***	0.5	0.3	***3.1***	***7.9***	1.5	0.5	***7.4***	***14***	***6.7***	1.5	***7.5***	***7.4***	***11***	***15***
IFI27	Interferon alpha-inducible protein 27	Hs. 278613	***6.6***	***4.4***	0.6	0.3	***3.5***	***7.1***	1.5	0.6	***6.6***	***14***	***6.6***	1.4	***6.7***	***6.6***	***11***	***14***
OAS3	2′-5′oligoadenylate synthetase 3	Hs. 56009	***5.5***	***5.3***	1.4	0.5	***3.2***	***2.3***	1.1	0.8	***4.5***	***4.5***	2.0	1.1	***4.7***	***4.5***	***4.8***	***4.5***
GIP3	Interferon alpha-inducible protein (clone IFI-6-16)	Hs. 265827	***4.0***	***3.8***	0.7	0.3	0.8	***5.3***	2.2	0.6	***4.3***	***5.0***	2.5	1.4	***5.8***	***4.3***	***4.7***	***5.0***
IFIT1	Interferon-induced protein w/tetratricopeptide repeat	Hs. 20315	***3.8***	***2.8***	0.6	0.9	***3.2***	***6.4***	0.9	0.5	***4.3***	***4.9***	***6.6***	0.6	***6.8***	***4.3***	***5.3***	***4.9***
MX1	Myxovirus (influenza) resistance 1	Hs. 48516	***3.6***	***3.8***	1.0	0.7	***3.1***	***2.8***	1.0	1.5	***3.4***	***2.8***	1.8	0.8	***4.2***	***3.4***	***3.7***	***2.8***
B2M	Beta-2-microglobulin	Hs. 75415	***3.2***	***2.2***	1.4	2.0	0.8	***3.9***	2.4	0.6	***2.3***	***2.2***	2.2	1.2	***2.5***	2.3	***2.4***	***2.2***
OAS2	2′-5′oligoadenylate synthetase 2	Hs. 432659	***2.7***	***2.2***	1.0	0.8	2.0	***2.0***	1.1	1.1	***3.2***	***2.2***	2.1	1.3	1.1	1.3	0.9	1.1
SP110	Interferon-induced protein 75 (52 kD)	Hs. 38125	***2.7***	***2.3***	1.3	0.7	1.4	0.9	1.3	0.8	1.8	1.3	1.3	1.3	***3.3***	***3.2***	***2.4***	***2.2***
Cellular Immune Response																		
ILF3	Interleukin enhancer binding factor 3, 90 kD	Hs. 56583	***3.0***	***2.3***	1.6	0.8	1.5	0.9	1.7	1.1	1.3	1.1	0.7	1.1	1.1	1.3	0.9	1.1
TIA1	Cytotoxic granule-associated RNA-binding protein	Hs. 39489	***2.6***	***2.2***	2.1	1.0	1.3	0.6	1.1	1.1	1.2	1.2	0.7	2.0	1.2	1.2	1.4	1.2
Cell Growth/Signal Transduction																		
FOSB	FBJ murine osteosarcoma viral oncogene homolog B	Hs. 75678	***10***	***5.4***	0.4	0.3	0.7	0.4	0.4	0.7	1.2	0.6	0.6	0.6	0.8	1.2	1.1	0.6
SMARCB	Actin dependent regulator of chromatin (SWI/SNF related)	Hs. 59971	***7.9***	***4.5***	0.6	0.5	1.1	1.1	1.0	0.9	1.7	0.8	0.9	0.8	1.5	1.7	1.3	0.8
ITGA6	Integrin alpha 6	Hs. 27730	***6.6***	***3.4***	1.4	0.6	0.7	1.2	1.3	1.3	1.7	1.3	1.3	1.2	1.9	1.7	***1.9***	1.3
JUN	v-jun avian sarcoma virus 17 oncogene homolog	Hs. 78465	***4.7***	***3.3***	0.3	0.6	0.9	0.9	1.0	0.9	1.5	0.6	0.6	0.9	0.8	1.5	0.7	0.6
PRNP	Prion protein (p27–30)	Hs. 74621	***3.6***	***2.1***	2.1	2.0	0.7	1.7	1.3	0.6	1.4	0.5	1.0	0.8	1.1	1.4	0.7	0.5
ID2	Inhibitor of DNA binding 2	Hs. 180919	***3.1***	***2.2***	0.5	0.6	0.6	0.9	0.7	0.8	2.1	1.3	1.2	1.3	***2.4***	2.1	1.7	1.3
JUNB	Jun B proto-oncogene	Hs. 400124	***3.0***	***2.8***	1.7	1.3	1.1	0.8	1.0	1.3	2.3	***2.4***	0.9	1.3	1.8	2.3	1.6	***2.4***
TOB2	Transducer of ERBB2	Hs. 4994	***2.8***	***2.3***	1.9	0.6	1.9	1.4	1.7	2.3	2.1	1.6	1.2	1.7	***2.6***	2.1	1.7	1.6
PRSS21	Serine protease, 21 (testisin)	Hs. 72026	***2.5***	***2.5***	1.6	1.0	2.3	***2.2***	1.8	1.7	***2.7***	***2.6***	***2.9***	1.4	2.1	***2.7***	***2.6***	***2.6***
TRB2	GS3955 protein	Hs. 55418	***2.1***	***2.2***	1.8	1.1	1.0	1.5	1.7	1.6	1.9	1.5	1.5	1.5	1.2	1.9	1.5	1.5
ARHD	Ras homolog gene family, member	Hs. 15114	***2.0***	***2.4***	0.8	0.8	0.9	0.8	0.9	1.1	1.3	0.9	0.9	0.9	0.9	1.3	1.1	0.9
	Confidence interval		1.9	2.1	2.2	2.4	2.5	2.0	2.6	2.4	2.5	2.0	2.6	2.4	2.2	2.5	1.9	2.0

a Expression levels of genes induced above the confidence interval at weeks 4 & 6 but below the confidence interval at weeks 13 & 40 in X0190.

The bold and italicized values represent data above the 99% confidence interval as described in the text.

### Comparison of hepatic gene expression during self-limited and persistent infections

To determine whether a different pattern of gene expression is observed between infected chimpanzees in this subset of genes, biopsy samples from two persistently infected chimpanzees X0234 and X0142 were compared to chimpanzee X0190 (Table 1). Within the first 8 weeks of infection, a large number of type I interferon stimulated genes (ISGs) were strongly up-regulated in all chimpanzees. Type I interferon induced genes are the first line of innate defense against viral infection and also function to prime and modulate adaptive immune response. A qualitative or quantitative difference of ISG induction could not be correlated with persistence or clearance. However several genes related to cellular immune response and cell growth/signal transduction, such as ILF3, TIA1, FOSB, JUN, ID2, were differentially induced in chimpanzee X0190.

**Figure 1:**
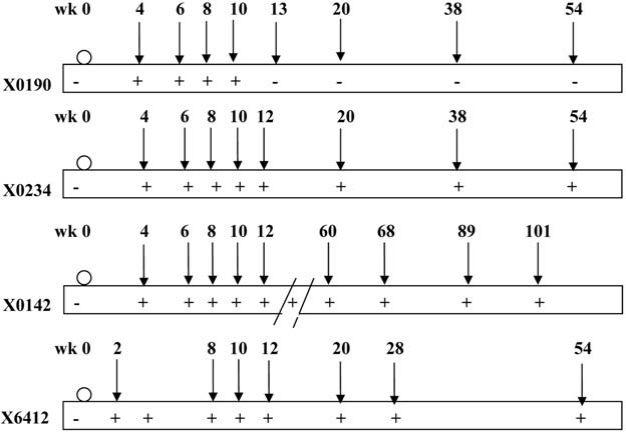
Chimpanzee inoculation and biopsy schedule. Chimpanzees were inoculated with RNA transcript of the molecular clone HCV-CG1b or infectious serum from the same clone (○). The course of infection has been described previously (Thomson et al., 2001). The level of viremia is shown as either positive (+) negative (−). Biopsy samples were taken at various time points after inoculation (↓) and used for microarray analysis X0190, X0234 and X0142). RNA samples from weeks 8 and 10 of X0190 were not of adequate quality for microarray analysis.

At later time points for X0190, none of the type I interferon response genes were significantly upregulated, consistent with the clearance of viremia and resolution of infection. Interestingly, a similar gene expression pattern was also observed for the persistently infected chimps. In X0234, some of these genes that were induced earlier returned to baseline at week 10, but became elevated again at week 12 post-infection. It has been shown that acutely infected chimpanzees and humans can have markedly fluctuating levels of viremia during the acute phase, possibly indicating transient control of viral infection [9]–[11]. This transient control could explain the lack of continuous induction of ISGs at certain time points. In particular, the viral level of X0234 at week 8 was 4000 genomes/mL, week 10 was 600 genomes/mL (borderline detection), and week 12 was 7000 genomes/mL. Additional biopsy samples from one of these animals, X0142, were collected at much later time points during the chronic phase (Fig. 1). Up-regulation of many ISGs was again observed, suggesting the establishment of chronic infection. This observation is consistent with previous publications [3], [6].

As an alternative approach to identify genes induced during the early stage of infection that could be associated with clearance, the average expression profile of persistently infected chimpanzees (X0234 and X0140) was compared to the average expression profile of chimp X0190 during weeks 4 and 6. We reasoned that any difference between the two groups of animals, if significant, might be more easily detected by averaging the gene expression values during this time frame. Furthermore, we might identify a different set of induced genes compared to our previous analysis (e.g. genes whose fold-induction levels were within the confidence interval for at least one time point but were on average higher during the acute phase of infection).

During the first eight weeks of self limited infection (X1090) we found 36 known genes induced 2.5-fold or greater over those during persistent infection (X0234 and X0140). The averaged ratios of induction are shown in Table 2. Six of these genes were also found on the list of the previous analysis of chimpanzee X0190 at weeks 4 and 6 (Table 1).

**Table 2:** Genes induced during the early phase of self-limited infection as defined by average expression values.

Title	Clone description	UG cluster	Self-limited infection^a^	Persistent infection^a^	Ratio of induction
**FOSB**	**FBJ murine osteosarcoma viral oncogene homolog B**	**Hs.75678**	**7.54**	**0.77**	**9.77**
DCT	Dopachrome tautomerase (dopachrome delta-isomerase, tyrosine-related protein 2)	Hs.301865	6.55	0.69	9.44
**SMARCB**	**Actin dependent regulator of chromatin (SWI/SNF related)**	**Hs.159971**	**6.04**	**0.64**	**9.41**
EGR1	Early growth response 1	Hs.738	2.50	0.30	8.20
ETR101	Immediate early protein	Hs.737	8.02	1.19	6.75
RPS4Y	Ribosomal protein S4, Y-linked	Hs.180911	8.46	1.40	6.02
**JUN**	**v-jun avian sarcoma virus 17 oncogene homolog**	**Hs.78465**	**3.99**	**0.76**	**5.21**
CYP1A1	Cytochrome P450, subfamily I (aromatic compound-inducible), polypeptide 1	Hs.72912	2.18	0.48	4.51
SCYA3	Small inducible cytokine A3 (homologous to mouse Mip-1a)	Hs.73817	1.23	0.29	4.28
PEX1	Peroxisome biogenesis factor 1	Hs.99847	3.68	0.90	4.09
KIAA0855	Golgin-67	Hs.182982	2.01	0.54	3.75
SARS	Seryl-tRNA synthetase	Hs.4888	1.13	0.31	3.68
GLO1	Glyoxalase I	Hs.75207	4.14	1.14	3.63
**ITGA6**	**Integrin alpha 6**	**Hs.227730**	**4.79**	**1.35**	**3.55**
DUSP1	Dual specificity phosphatase 1	Hs.171695	2.31	0.68	3.41
**PRNP**	**Prion protein (p27–30)**	**Hs.74621**	**2.79**	**0.87**	**3.21**
RUVBL2	RuvB (E coli homolog)-like 2	Hs.6455	1.80	0.58	3.10
SCYA3L1	Small inducible cytokine A3-like 1	Hs.274535	0.97	0.31	3.10
TRF4	Topoisomerase-related function protein 4-1	Hs.225951	1.73	0.57	3.03
CALML3	Calmodulin-like 3	Hs.239600	2.96	1.01	2.94
GCN1L1	GCN1 (general control of amino-acid synthesis 1, yeast)-like 1	Hs.75354	1.58	0.55	2.89
SCD	Stearoyl-CoA desaturase (delta-9-desaturase)	Hs.119597	1.16	0.40	2.88
ATP2B2	ATPase, Ca++ transporting, plasma membrane 2	Hs.89512	1.40	0.49	2.86
CYP4F3	Cytochrome P450, subfamily IVF, polypeptide 3 (leukotriene B4 omega hydroxylase)	Hs.106242	2.94	1.05	2.80
COL7A1	Collagen, type VII, alpha 1 (epidermolysis bullosa, dystrophic, dominant and recessive)	Hs.1640	1.58	0.57	2.78
SLC6A3	Solute carrier family 6 (neurotransmitter transporter, dopamine), member 3	Hs.406	2.57	0.95	2.70
KLF4	Kruppel-like factor 4 (gut)	Hs.7934	2.23	0.83	2.67
**ILF3**	**Interleukin enhancer binding factor 3, 90 kD**	**Hs.256583**	**2.63**	**1.00**	**2.62**
**ID2**	**Inhibitor of DNA binding 2**	**Hs.180919**	**2.64**	**1.02**	**2.58**
ZNF216	Zinc finger protein 216	Hs.3776	1.07	0.41	2.57
NR1I2	Nuclear receptor subfamily 1, group I, member 2	Hs.118138	1.89	0.74	2.56
**TIA1**	**TIA1 cytotoxic granule-associated RNA-binding protein**	**Hs.239489**	**2.40**	**0.94**	**2.56**
CDK9	Cyclin-dependent kinase 9 (CDC2-related kinase)	Hs.150423	1.66	0.66	2.52
RELA	v-rel avian reticuloendotheliosis viral oncogene homolog A (nuclear factor of kappa light polypepti	Hs.75569	1.79	0.71	2.52
EPHX2	Epoxide hydrolase 2, cytoplasmic	Hs.113	2.50	1.00	2.51
CYP4F2	Cytochrome P450, subfamily IVF, polypeptide 2	Hs.101	1.99	0.79	2.51

a Average expression values of biopsies taken during the 1^st^ 8 weeks of infection.

Bold-face genes are also in Table 1.

### Hepatic gene expression during the clearance phase of self-limited infection

To identify genes that may be specifically induced during viral clearance in chimpanzee X0190, the database of expression levels was queried for genes above the confidence interval at 13 weeks post-infection (>2.2-fold induction) and below this interval at 4, 6 and 40 weeks (<1.9-, 2.1-, and 2.4-fold induction). Forty-four genes were significantly up-regulated at 13 weeks (Table 3). Twenty of these were associated with activation of cellular immunity and as such were classified as Cellular Immune Response-related. Consistent with the previous observation of active HCV-specific T-cell response in biopsy specimens during resolving infections, we found evidence for a vigorous immune response involving induction of CD8+ T-cell markers (CD8 antigen and components of the T-cell receptor) and effectors of CD8+ T cells (granzyme A and interferon-gamma) in X0190. The remaining 25 genes significantly induced at 13 weeks could be classified as Cell Growth/Signal Transduction-related (Table 3).

**Table 3:** Genes induced during the clearance phase of self-limited infection.

Cellular Immune Response			Chimp X0190^a^				Chimp X0234				Chimp X0142							
Title	Clone description	UG cluster	wk 4	wk 6	wk 13	wk 40	wk 6	wk 8	wk 10	wk 12	wk 6	wk 8	wk 10	wk 12	wk 60	wk 68	wk 89	wk 101
GZMA	Granzyme A	Hs.90708	1.3	1.7	***6.5***	1.5	1.2	2.5	1.8	1.7	***5.6***	1.3	2.5	***4.7***	1.7	***5.6***	***2.2***	1.3
CD3D	CD3D antigen, delta polypeptide	Hs.95327	1.7	2.0	***6.4***	2.0	1.3	***2.7***	***2.4***	1.7	***3.9***	1.2	1.7	***3.6***	***2.5***	***3.9***	***2.1***	1.2
IGL	Immunoglobulin lambda locus	Hs.05944	1.7	2.1	***5.8***	2.0	***3.2***	***3.3***	***6.0***	***4.2***	***2.5***	***5.0***	1.4	***3.9***	1.7	***2.5***	***5.6***	***5.0***
CD8B1	CD8 antigen, beta polypeptide 1 (p37)	Hs.2299	1.4	1.8	***5.6***	1.3	1.3	1.9	***2.0***	1.6	1.6	1.4	1.2	1.8	1.3	1.6	1.6	1.4
CCL5	Small inducible cytokine A5 (RANTES)	Hs. 41392	0.9	1.1	***4.8***	1.0	1.3	1.7	***2.7***	2.8	2.7	***2.4***	1.0	2.2	***2.7***	***2.7***	***2.2***	***2.4***
TRA	Human T-cell receptor active alpha-chain	Hs.74647	1.8	1.7	***4.5***	1.4	1.9	***5.1***	***4.2***	3.1	2.6	***2.4***	***3.0***	***4.2***	1.8	***2.6***	***3.3***	***2.4***
CSF2RA	Colony stimulating factor 2 receptor	Hs. 82378	1.3	1.9	***4.0***	1.7	***2.4***	1.4	***3.1***	***4.3***	1.9	***2.8***	1.1	***3.2***	***3.3***	1.9	***2.1***	***2.8***
KLRB1	Killer cell lectin-like receptor subfamily B	Hs.69824	1.9	2.1	***3.9***	2.1	1.0	2.6	1.6	1.1	***3.6***	1.0	1.6	0.9	***2.5***	***3.6***	1.7	1.0
CD8A	CD8 antigen, alpha polypeptide (p32)	Hs.85258	1.6	1.4	***3.5***	1.4	0.8	1.6	1.7	1.4	2.0	1.1	0.8	1.5	1.6	2.0	1.1	1.1
IGHG3	Immunoglobulin heavy constant gamma 3	Hs.413826	1.6	1.6	***3.4***	1.0	***2.5***	2.3	***3.2***	***3.3***	***2.6***	***2.8***	1.0	***3.0***	1.8	***2.6***	***5.0***	***2.8***
CD2	CD2 antigen (p50)	Hs.89476	1.9	1.3	***3.3***	1.4	1.3	***5.6***	***4.0***	2.0	***2.6***	1.9	2.0	***3.3***	***3.0***	***2.6***	***2.4***	1.9
TRGV9	T cell receptor gamma locus	Hs.12259	1.2	1.9	***3.0***	1.8	1.6	***3.2***	***3.2***	1.6	***2.8***	1.8	1.5	1.6	1.6	***2.8***	***2.2***	1.8
ITK	IL2-inducible T-cell kinase	Hs.11576	1.7	1.7	***3.0***	1.2	1.1	2.6	***2.2***	1.4	1.7	1.6	1.6	1.6	1.9	1.7	1.5	1.6
SELPLG	Selectin P ligand	Hs.79283	1.5	1.6	***2.8***	1.3	1.6	***2.9***	***2.8***	3.0	***2.6***	1.7	1.2	***2.6***	***2.7***	***2.6***	1.5	1.7
VIL2	Villin 2 (ezrin)	Hs.155191	1.1	1.8	***2.4***	1.5	1.8	1.4	***2.8***	2.2	2.3	***2.2***	1.4	1.4	1.9	2.2	2.3	1.3
RI58	Retinoic acid- and interferon-inducible protein (58 kD)	Hs.27610	0.7	0.9	***2.4***	0.7	0.8	0.6	0.8	0.7	0.8	0.7	0.7	0.5	0.8	0.8	0.8	0.7
IFNG	Interferon gamma	Hs.856	1.2	1.6	***2.4***	1.3	1.1	1.5	1.6	1.2	1.8	1.1	1.1	1.3	1.6	1.8	1.7	1.1
TANK	TRAF family member-associated NFKB activator	Hs.146847	1.3	1.5	***2.3***	2.1	1.5	1.5	1.5	1.5	1.5	1.1	1.6	1.3	1.6	1.5	1.7	1.1
TNFRSF1A	Tumor necrosis factor receptor (1A)	Hs.159	1.6	1.2	***2.3***	1.1	***3.7***	0.6	1.9	***3.6***	1.1	1.5	0.6	1.5	1.2	1.1	1.4	1.5
PADI4	Peptidyl arginine deiminase (type IV)	Hs.117232	0.8	1.3	***2.2***	1.7	1.7	1.1	1.1	2.4	2.0	1.7	1.2	1.2	1.6	2.0	1.3	1.7
Cell Growth/Signal Transduction																		
SFTPA2	Surfactant (pulminory-associated protein A1)	Hs.177582	1.2	1.4	***3.6***	1.2	1.9	1.5	1.8	2.8	2.0	1.9	1.1	1.7	1.8	2.0	1.3	1.9
UBD	Diubiquitin	Hs.44532	0.9	0.6	***3.3***	0.5	1.8	***4.5***	***3.4***	2.9	2.3	1.4	1.2	1.5	1.5	2.3	1.4	1.4
HOXB5	Homeo box B5	Hs.22554	1.1	1.7	***3.2***	1.6	1.9	1.1	***2.5***	3.2	1.1	***2.6***	0.9	2.0	***2.3***	1.1	1.8	1.6
BAZ2B	Bromodomain adjacent to zinc finger domain, 2B	Hs.8383	1.6	1.2	***3.0***	0.8	1.4	1.0	0.9	2.0	1.0	0.9	0.8	1.4	1.2	1.0	1.0	0.9
AF1Q	ALL1-fused gene from chromosome 1q	Hs.75823	1.8	2.0	***2.6***	2.1	***4.7***	***4.6***	***2.4***	2.8	***4.5***	***2.4***	***3.9***	***2.5***	2.1	***4.5***	***2.3***	***2.2***
ITM2A	Integral membrane protein 2A	Hs.17109	1.0	1.4	***2.6***	1.7	1.1	1.8	1.3	1.5	***2.7***	1.5	1.3	2.3	1.6	***2.7***	1.5	1.5
ANXA6	Annexin A6	Hs.118796	1.0	1.4	***2.5***	1.4	***2.2***	2.0	2.0	***5.0***	***3.3***	1.3	0.9	2.3	***2.8***	***3.3***	***2.0***	1.3
SFRS3	Splicing factor, arginine/serine-rich 3	Hs.388623	1.8	1.8	***2.5***	1.7	***2.4***	2.1	1.9	2.9	1.5	0.9	1.5	2.1	2.1	1.5	1.2	0.9
AMY2A	Amylase, alpha 2A; pancreatic	Hs.300280	1.2	1.6	***2.5***	1.5	1.5	0.9	1.2	1.9	0.8	1.2	0.4	1.0	1.3	0.8	0.8	1.2
KIF22	Kinesin-like 4	Hs.119324	1.4	0.9	***2.5***	1.7	***2.5***	***3.3***	***2.1***	***3.9***	1.3	1.1	***2.6***	2.2	1.2	1.3	1.2	1.1
CAPON	Ligand of neuronal nitric oxide synthase	Hs.129729	1.7	1.6	***2.5***	1.2	***2.5***	0.4	0.8	2.3	1.3	***2.0***	0.5	***2.6***	1.4	1.3	***2.1***	***2.0***
COX5B	Cytochrome c oxidase (subunit Vb)	Hs.1342	1.4	1.0	***2.5***	1.8	1.2	***3.8***	1.7	1.7	1.6	1.0	2.4	1.2	1.9	1.6	1.7	1.0
LGALS1	Galactoside-binding lectin (galectin 1)	Hs.382367	1.6	1.2	***2.4***	2.0	1.3	***3.3***	***2.9***	1.3	1.9	1.0	1.4	1.3	***2.5***	1.9	***2.3***	1.0
MBL1P1	Mannose-binding lectin, pseudogene 1	Hs.116218	1.7	1.6	***2.4***	1.6	1.2	0.6	0.8	1.1	1.4	***2.4***	0.8	2.1	1.1	1.4	1.3	***2.4***
GNG3	Guanine nucleotide binding protein	Hs.179915	1.5	1.4	***2.4***	2.1	1.7	1.7	***2.4***	2.9	1.7	1.2	1.3	2.3	2.0	1.7	1.8	1.2
RBBP4	Retinoblastoma-binding protein 4	Hs.16003	1.2	1.3	***2.4***	1.7	1.2	1.1	1.0	1.7	1.7	1.2	1.7	1.2	1.4	1.7	1.4	1.2
CHI3L1	Chitinase 3-like 1 (cartilage glycoprotein-39)	Hs.75184	0.9	1.6	***2.4***	1.9	1.5	1.5	1.1	2.7	1.2	1.6	0.9	0.9	***2.3***	1.2	1.1	1.6
NESG1	Nasopharyngeal epithelium specific protein 1	Hs.158450	0.6	1.5	***2.3***	1.6	1.1	1.1	0.8	3.2	1.2	1.2	0.7	1.1	***2.5***	1.2	1.0	1.2
CBLC	Cas-Br-M Murine retroviral transforming	Hs.156637	0.6	1.5	***2.3***	1.6	1.2	0.9	0.8	***3.7***	1.5	1.4	1.2	1.1	***3.8***	1.5	1.8	1.4
SLC21A6	Solute carrier family 21	Hs.137425	1.4	1.1	***2.3***	1.1	***3.5***	0.6	1.8	***4.3***	1.1	1.3	0.5	1.4	1.0	1.1	1.2	1.3
DSS1	Deleted in split-hand/split-foot 1 region	Hs.333495	1.2	1.0	***2.3***	1.5	1.2	1.2	1.2	1.1	1.8	1.2	2.2	1.0	1.4	1.8	1.5	1.2
BAIAP1	BAI1-associated protein 1	Hs.169441	1.1	1.3	***2.3***	1.2	1.2	***8.0***	1.1	1.4	1.8	***2.0***	0.7	1.4	1.3	1.8	1.3	***2.0***
PCAF	PCAF associated factor 65 beta	Hs.26782	1.7	1.7	***2.2***	1.3	***2.2***	2.4	***2.8***	2.1	1.3	0.9	0.8	1.1	1.1	1.3	0.7	0.9
EIF2AK3	Eukaryotic translation initiation factor 2-alpha kinase 3	Hs.102506	1.5	1.4	***2.2***	1.3	1.0	0.9	0.8	1.1	1.3	1.1	0.8	2.1	1.0	1.3	1.2	1.1
	99% confidence interval		1.9	2.1	2.2	2.4	2.1	2.7	2.0	3.3	2.5	2.0	2.6	2.4	2.2	2.5	1.9	2.0

a Expression levels of genes induced above the confidence interval at week 13 but below the confidence interval at weeks 4, 6 & 40 of X0190.

The bold and italicized values represent data above the 99% confidence interval as described in the text.

To discern additional differences between the self-limited and chronically infected chimpanzees, the averaged expression profiles of the two persistently infected chimpanzees (X0142 and X0234) from weeks 10 to 12 were compared to those of chimpanzee X0190 during clearance (week 13). We reason that viral clearance may be associated with a distinct set of gene expression in the liver. By comparing the gene expression profiles at about the same time when the chimpanzee is undergoing viral clearance to those of chimpanzee who is not could provide valuable insight into the mechanism of viral clearance. During this phase, 20 known genes of X0190 were induced 2.5-fold or greater over the average values of the equivalent time points of X0142 and X0234 (Table 4). Of these, 5 genes are also in Table 3.

**Table 4:** Genes induced during the clearance phase of self-limited infection as defined by average expression values.

Title	Clone description	UG cluster	Self-limited infection^a^	Persistent infection^b^	Ratio of induction
RPS4Y	Ribosomal protein S4, Y-linked	Hs.180911	12.66	1.64	7.72
GLO1	Glyoxalase I	Hs.268849	4.07	0.75	5.41
NPTX1	Neuronal pentraxin I	Hs.84154	1.45	0.40	3.59
**RI58**	**Retinoic acid- and interferon-inducible protein (58 kD)**	**Hs.252839**	**2.40**	**0.70**	**3.42**
UK114	Translational inhibitor protein p14.5	Hs.18426	2.00	0.59	3.40
AMHR2	Anti-Mullerian hormone receptor, type II	Hs.437877	0.97	0.30	3.19
**CD8B1**	**CD8 antigen, beta polypeptide 1 (p37)**	**Hs.2299**	**5.57**	**1.76**	**3.16**
**KLRB1**	**Killer cell lectin-like receptor subfamily B, member 1**	**Hs.169824**	**3.89**	**1.24**	**3.13**
APOF	Apolipoprotein F	Hs.2388	1.00	0.32	3.12
KIAA0855	Golgin-67	Hs.182982	1.60	0.54	2.95
TSNAX	Translin-associated factor X	Hs.96247	1.81	0.63	2.89
FKBP5	FK506-binding protein 5	Hs.7557	2.69	0.94	2.87
NNMT	Nicotinamide N-methyltransferase	Hs.364345	2.07	0.76	2.73
NUP153	Nucleoporin 153 kD	Hs.146449	1.95	0.72	2.71
SOCS3	Supressor of cytokine signaling 3	Hs.436943	2.54	0.94	2.69
**CD3D**	**CD3D antigen, delta polypeptide**	**Hs.95327**	**6.44**	**2.42**	**2.66**
CALML3	Calmodulin-like 3	Hs.239600	3.37	1.28	2.63
ARF3	Human ADP-ribosylation factor	Hs.22012	1.40	0.54	2.58
**GZMA**	**Granzyme A (granzyme 1, cytotoxic T-lymphocyte-associated serine esterase 3)**	**Hs.90708**	**6.48**	**2.56**	**2.53**

a Expression value of biopsy taken at 13 weeks of infection.

b Average expression values of biopsies taken during the 10 and 12 weeks of infection.

Bold-face genes are also in Table 1.

### Quantitative PCR confirmation of selected genes

To confirm the microarray data, TaqMan real-time PCR was performed on selected genes from each analysis. For ISGs, GIP2 and IFIT1 were analyzed. Among the genes identified in Tables 1 and 2, ILF3, TIA1, ID2 and JUN were selected for quantitative PCR. In addition, serial liver biopsy RNAs from another chimpanzee that developed chronic infection after inoculation with a different HCV strain (H77 1a) were analyzed. The data in general support the microarray results. The ISG expression varied among the chimpanzees, but showed no significant difference between self-limited and chronic infections. The genes (ILF3, TIA1, ID2 and JUN) identified to be different by microarray during the early phase of HCV infection between the two groups were confirmed by quantitative PCR. Their expression levels were 10 to 100-fold higher in X0190 than those of other three chimpanzees.

## Discussion

HCV infection can lead to a high rate of chronicity, with 70–80% of infected persons developing persistent infection [1]. The mechanisms by which HCV establishes chronic infection have been the subject of intense research. Failure of HCV-specific immune response, particularly of the T cells, has been proposed as the cause of chronicity [12], [13]. Studies in humans and chimpanzees have shown that T cell-mediated immunity is important for viral clearance [14], [15]. However, the pivotal question remains as to how the host immune response fails during the acute HCV infection so it can no longer control the virus, resulting in persistent infection. Thus it is crucial to define the molecular and cellular mechanisms by which the antiviral host response is activated and regulated during the early stage of acute HCV infection. Furthermore, because the major site of viral tropism is the liver, it is essential to study these events in the liver. One approach to elucidate this complicated process is to study the global gene expression profile in the liver during the acute phase of viral infection, and to discern unique patterns that are associated with either viral clearance or progression to chronic infection.

We previously reported the infection of three chimpanzees with an infectious HCV genotype 1b clone; one had acute self-limited infection and the other two developed chronic infection [16]. We reported that regardless of the outcome of infection, peripheral T-cell responses were weak and comparable among the chimpanzees during the course of infection; however, intrahepatic T-cell response was not analyzed [17]. In this study, serial liver biopsies were available in these chimpanzees and were used for cDNA microarray analysis. Analysis of these gene expression patterns revealed that a type I interferon response was induced during the early phase (4–8 weeks) of infection in all chimpanzees regardless of the outcome of infection. Various well-defined interferon stimulated genes were up-regulated in all the liver samples. This observation is consistent with previous studies describing that type I interferon response is rapidly induced in the liver in response to HCV infection [2], [6].

**Figure 2:**
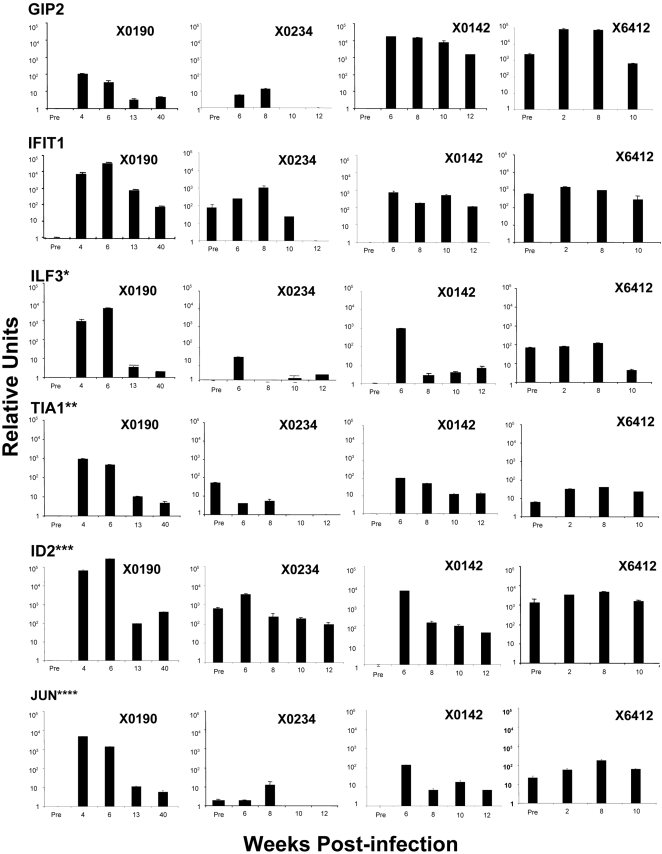
Real-time PCR quantification of candidate genes involved in viral clearance and persistence. TaqMan real-time PCR was performed as described in Materials and Methods. The y-axis shows the relative unit of a given gene normalized to GAPDH and 18s rRNA. Data are expressed as means±SEM. In all cases, average values obtained during the first eight weeks of infection were compared between recovered (X0190) and chronically infected chimpanzees (X 0234, X0142 and X6412). * *P<0.05*, ** *P<0.01*, *** *P<0.01*, **** *P<0.005*.

Interestingly, several genes were specifically induced in the recovered chimpanzee during the early phase of infection but not in those chimpanzees with chronic infection. Two of the genes are interleukin enhancer binding factor 3 (ILF3) and cytotoxic granule-associated RNA binding protein (TIA1). This difference was also confirmed by quantitative RT-PCR (Fig. 2). Both of them are related to cellular immune response and may potentially herald the emergence of a robust T-cell response later. The other genes could be functionally clustered into cell growth/signal transduction pathways. Genes such as FOSB, JUN, JUNB, and ID2 (Tables 1 and 2) are typically associated with the immediate early genes during liver regeneration [18], [19]. Several studies reported that ID2 (Inhibitor of DNA binding or Differentiation) protein, a helix-loop-helix transcription factor, has important roles in cell growth, differentiation and angiogenesis [20], [21]. ID2 is also essential for NK lineage commitment from bipotent progenitors of both T and NK cells [22]. Other up-regulated genes including EGR1, ETR101, CDK9 and RelA, are also related to cell growth and proliferation [23]–[25]. Induction of EGR1 and ETR101 is also part of the early proliferative response in the liver. In addition, EGR1 is involved in the induction of FasL in T cells [26] and the ETR101 has been implicated in T cell proliferation and maturation [27]. These two genes might represent the induction of a successful T cell response against HCV in chimpanzee X0190 but not in X0142 and X0234. The induction of these genes may signify the initial phase of hepatocyte injury and proliferation, despite the absence of aminotransferase elevation and liver pathology during this stage of infection. This observation has implication with respect to the induction of adaptive immunity that is important for the subsequent control of viral infection. This pattern of gene expression in the liver may represent a successful “danger signal” that has been proposed to be a trigger for adaptive immunity. The lack of induction of these genes in chimpanzees that progress to chronic infection may actually represent a failure to amplify anti-viral T-cell response. This intriguing hypothesis awaits further studies to clarify the role of these genes during HCV infection.

During acute HCV infection, a rapid IFN response limits virus replication and spread in the liver until virus-infected hepatocytes are cleared by specific T-cell immune response [3]. Analysis of gene expression patterns during the viral clearance phase of chimpanzee X0190 reveals the intrahepatic induction of cellular immune response. This is particularly evident with markers of CD8+ T cell response including granzyme A, CD8 antigen, T cell receptors, and interferon-gamma (Table 3). On the other hand, fewer of these genes were induced and at a lower magnitude in chimpanzees with chronic infection. These findings are consistent with a recent study on hepatic gene expression in chimpanzees during acute HCV infection [2], [6], and support the importance of T cell immune response in controlling HCV infection [28]–[33], [12], [34], [7]. Other genes that were preferentially induced during the clearance phase are diverse. They do not overlap with those genes induced during the early phase of infection and may represent a more complex array of molecular and cellular events during viral clearance. All these genes returned to baseline expression after viral clearance. In contrast, many ISGs remained elevated in chimpanzees with chronic infection, indicating an ongoing type I IFN response to persistent HCV infection.

In this study, molecular profiling of gene expression patterns in chimpanzees with serial liver biopsies during the course of infection provides valuable information on the potential mechanisms of viral clearance and persistence. Although the number of animals is small, we are able to define a unique gene expression pattern associated with viral clearance and demonstrate the potential importance of induction of certain genes in a “successful” anti-HCV response. IFN-stimulated genes (ISGs) are induced similarly regardless of the outcome of infection. Early induction of a set of genes associated with cell proliferation and immune activation appears to be involved in subsequent viral clearance. Furthermore, evidence for a strong intrahepatic induction of cellular immune response in chimpanzees associated with self-limited infection is present. These findings support the importance of T-cell immune response in controlling HCV infection. Additional studies in other chimpanzees or humans with well-characterized course of acute infection are necessary to confirm these observations.

## Materials and Methods

### Animals

Chimpanzees *(Pan troglodyte)* were housed at the Southwest Foundation for Biomedical Research, an Association for Assessment and Accreditation of Laboratory Animal and Care (AAALAC)-accredited facility, and the study protocol was approved by the Institutional Animal Care and Use Committee at the Foundation and by the Interagency Animal Model Committee at the National Institutes of Health. Three chimpanzees (X0190, X0142 and X0234) were infected with HCV CG1b strain, either by intrahepatic inoculation of HCV RNA or HCV-positive serum, as described previously [16]. One animal X0190 recovered from the infection and the other two (X0142 and X0234) developed chronic infection. The infection courses of all three chimpanzees have been described in detail previously [16], [17] and summarized in Fig. 1 with time points of liver biopsy. Another chimpanzee X6412 was infected with H77 1a strain and developed chronic infection [35]. Serial liver biopsies of this chimpanzee were provided by Stephen Feinstone of FDA.

### RNA extraction and microarray expression analysis

RNA was isolated from liver biopsies (about 20 mg of liver tissue which gives 10–15 µg of total RNA) of the chimpanzees. Deposition cDNA microarrays containing 8703 features were generated from IMAGE clones (ResGen, Huntsville, AL) as described previously [36], [37]. Liver biopsies were extracted directly with Trizol (Invitrogen, Carlsbad, CA) and Dounce homogenization. Following chloroform extraction, RNA samples were further purified with RNeasy columns (Qiagen, Valencia, CA). 5–20 µg total RNA was typically isolated from each liver biopsy and 5 µg of RNA was amplified using a RiboAmp protocol (Arcturus Bioscience Inc, Mountain View, California). A single round of amplification yielded approximately 60 µg of polyA-selected RNA, of which 2.5 µg was labeled by using the CyScribe first-strand cDNA and labeling protocol (Amersham/Pharmacia, Piscataway, New Jersey). Briefly, RNA was reverse transcribed to produce Cy-5 labeled cDNA while Cy-3 labeled cDNA was made in a similar manner from pre-infection or uninfected reference liver RNA. Individual Cy-5 labeled samples were mixed with the Cy-3 labeled reference and hybridized overnight at 65°C in an aqueous based hybridization solution. Detailed RNA isolation, labeling and hybridization protocols are available at http://research.nhgri.nih.gov/microarray/. Slide images were acquired by an Agilent scanner (Agilent Technologies, Palo Alto, CA). Gene assignments and expression data were extracted using the DeArray Suite [38] for IPLab spectrum. The resulting data was downloaded to FileMaker Pro (FileMaker Inc., Santa Clara, CA). Phenotype averaging was performed using the BRB array tools, developed by the Biometrics Research Branch, Division of Cancer Treatment and Diagnosis and available to download on the web: http://linus.nci.nih.gov/BRB-ArrayTools.html

### Real-time Quantitative PCR

Real-time quantitative PCR was used to confirm the microarray findings. Complementary DNA (cDNA) was synthesized from total RNA (isolated from liver biopsy samples) with First-strand cDNA Synthesis System (Marligen Biosciences, Ijamsville, MD). In addition, we analyzed serial liver biopsy samples from a chronically infected chimpanzee (X1602, infected with genotype 1a H77 strain) by TaqMan PCR quantification of selected genes of interest. The primers and probes used were obtained from Gene Expression Assays (Applied Biosystems, Foster City, CA). Each reaction was performed in duplicate, and all samples were standardized using the internal control glyceraldehyde-3-phosphate dehydrogenase (GAPDH) gene and 18S rRNA. Reactions were set up with 12.5 µL TaqMan universal PCR master mix, cDNA template, and 1.25 µL primers and probe mix in a final volume of 25 µL. Reactions were performed on an iCycler iQ Multicolor Real-Time Detection System (Bio-Rad, Hercules, CA) with the following reaction conditions: 95°C for 10 min, followed by 40 cycles of 95°C for 20 sec, 60°C for 1 min, and additional incubation at 68°C for 10 min.

### Statistical Analysis

The overall significance was assessed by 1-way ANOVA and significant difference between groups was assessed by the Student's *t*-test.
